# Misperception of sleep in bipolar disorder: an exploratory study using questionnaire *versus* actigraphy

**DOI:** 10.1186/s40345-020-00198-x

**Published:** 2020-11-20

**Authors:** Henrik Myhre Ihler, Manon Meyrel, Vincent Hennion, Julia Maruani, Gregory Gross, Pierre A. Geoffroy, Trine Vik Lagerberg, Ingrid Melle, Frank Bellivier, Jan Scott, Bruno Etain

**Affiliations:** 1 NORMENT, Division of Mental Health and Addiction, Oslo University Hospital & Institute of Clinical Medicine, University of Oslo, Oslo, Norway; 2 Assistance Publique des Hôpitaux de Paris, GHU Paris Nord, DMU Neurosciences, Hôpital Fernand Widal, Département de Psychiatrie et de Médecine Addictologique, Paris, France; 3 Faculté de Médecine, Université de Paris, Paris, France; 4 INSERM U1144, Optimisation Thérapeutique en Neuropsychopharmacologie, Paris, France; 5 Pôle de Psychiatrie et Psychologie Clinique, Centre Psychothérapique de Nancy, Laxou, France; 6 Faculté de Médecine, Université de Lorraine, Nancy, France; 7 Département de Psychiatrie et de médecine Addictologique, Assistance Publique des Hôpitaux de Paris (APHP), Centre Hospitalo-Universitaire Bichat-Claude Bernard, Paris, France; 8 Institute of Neuroscience, Newcastle University, Newcastle, UK

**Keywords:** Bipolar disorder, Sleep duration, Sleep latency, Sleep efficiency, Misperception of sleep

## Abstract

**Background:**

The concept of misperception of sleep refers to the estimated discrepancy between subjective and objective measures of sleep. This has been assessed only in a few prior studies in individuals with Bipolar Disorder (BD) as compared to Healthy Controls (HC) and with mixed results.

**Methods:**

We assessed a sample of 133 euthymic individuals with BD and 63 HC for retrospective subjective (Pittsburgh Sleep Quality Index) and objective (21 days of actigraphy recording) measures of total sleep time, sleep latency and sleep efficiency. We first investigated the correlations between these subjective and objective measures in the two groups. We then compared individuals with BD and HC for the absolute values of the differences between subjective and objective sleep parameters, used as a proxy of the magnitude of misperception of sleep. Finally, we undertook regression analyses to assess associations between clinical groups, core demographics, clinical factors and misperception of sleep.

**Results:**

The correlation coefficients between subjective and objective measures of sleep did not differ between groups (total sleep time: rho = .539 in BD and rho = .584 in HC; sleep latency: rho = .190 in BD and rho = .125 in HC; sleep efficiency: rho = .166 in BD and rho = .222 in HC). Individuals with BD did not differ from HC in the magnitude of misperception of total sleep time, sleep latency nor sleep efficiency. Individuals with BD type 1 misperceived their sleep efficiency significantly more than individuals with BD type 2, with no further difference between BD type 1 and BD type 2 regarding sleep latency and sleep duration misperceptions. Three factors (age, symptoms of obstructive sleep apnea, and mild depressive symptoms), were the main contributors to the magnitude of misperception of sleep.

**Conclusions:**

Misperception of sleep was not associated with a diagnosis of BD. In this sample, mild depressive symptoms, older age, or symptoms of obstructive sleep apnea may be related to greater sleep misperception. In that case, the reliability of subjective measures may decrease as the misperception of sleep increases. This study may help guide clinicians in selecting the best approach for assessing sleep (objective versus subjective measures) in individuals with BD.

## Background

Sleep disturbances are core symptoms of Bipolar Disorders (BD) (Ng et al. 2015; Geoffroy et al. 2015). In clinical practice, it is essential to screen for and manage sleep disturbances to improve affective and cognitive functioning, quality of life, metabolic health and to decrease relapse rates (Harvey et al. 2009; Gruber et al. 2009; Eidelman et al. 2010; Gruber et al. 2011; Etain et al. 2017; Brochard et al. 2018).

Clinicians can use different tools to assess sleep disturbances in terms of type, frequency and severity. The simplest form is to rely on the patients’ self-report of their sleep habits, perceived problems and spontaneous complaints. A more systematic approach includes the use of questionnaires, such as the Pittsburgh Sleep Quality Index (PSQI), which has demonstrated satisfactory validity and reliability in BD (Buysse et al. 1989). Questionnaires usually collect the information retrospectively, while sleep diaries can be used prospectively over a certain period of time. Both of these assessment tools are considered *subjective* measures of sleep disturbances and complaints.

In parallel, Sleep Polysomnography (PSG) provides an *objective* evaluation of sleep, and is currently considered the gold standard to assess sleep patterns. However, it is resource demanding, not widely available, and is often carried out in specialized laboratories. Actigraphy is also considered as an objective assessment of sleep/wake cycles. Actigraphy is less resource demanding than PSG, relatively inexpensive, and can easily be carried out in the patients’ home in an ecological manner, and with duration of recording ranging from days to months. The accuracy of actigraphy in measuring sleep parameters has been validated in individuals with BD (Baandrup and Jennum 2015; Sanchez-Ortuno et al. 2010).

As actigraphy has become widely used in BD, new possibilities have emerged to investigate *misperception of sleep*, i.e. estimation of discrepancies between subjective and objective measures of sleep in a given individual. This concept mainly emerged from studies in insomnia in which the individuals’ sleeping patterns were characterized by an overestimation of sleep onset latency (SL) and an underestimation of total sleep time (TST), relative to objective measures (Harvey and Tang 2012). A few previous studies in BD have investigated misperception of sleep using correlations between subjective sleep information (questionnaires or diaries) and objective sleep information (actigraphy), with the absence of correlation being an indicator of misperception. The results remain conflicting. Subjective (sleep diary) and objective (actigraphy) measures of total sleep time (TST) correlated well in a sample of 39 individuals with BD (Gonzalez et al. 2013). In a sample of 26 euthymic individuals with BD, subjective (PSQI data) and objective (actigraphy) measures of TST and SL correlated moderately well, while no correlation was observed for sleep efficiency (SE) (i.e. the ratio of the total time spent asleep (total sleep time) in a night compared to the total amount of time spent in bed) (Boudebesse et al. 2014). However, Krishnamurthy et al. did not find any correlation between subjective (PSQI) and objective (actigraphy) measures of TST or SL in 24 symptomatic individuals with BD (Krishnamurthy et al. 2018). In this context, correlations between subjective and objective measures of sleep can only be considered a proxy of misperception of sleep. Indeed, a low degree of correlation between subjective and objective measures is suggestive of misperception of sleep, while moderate correlations between measures do not definitively exclude the presence of misperception of sleep, and thus may be insufficient to draw any firm conclusions.

Therefore, misperception of sleep has been operationalized in two ways. The simplest way is to subtract the objective measure from the subjective measure or vice versa, and thus obtain a spectrum from −∞ to +∞. Values close to zero approximate no misperception, and values below or above a certain threshold can be considered as under- or overestimation of a given sleep parameter. Another option is to use the *absolute* value of misperception, where lower values represent less misperception, while both under- and overestimation correspond to higher values. The absolute value can here be considered a measure of the total *magnitude* of misperception of sleep, independent of direction.

The latter approach was used in the study by Krishnamurthy et al. (Krishnamurthy et al. 2018), who found that individuals with symptomatic BD had significantly higher misperception of sleep (TST and SL) compared to healthy controls (HC). Two independent studies, in addition to some prior studies in major depressive disorders (Rotenberg et al. 2000; Armitage et al. 1997), have suggested that the current mood states are potential major drivers of misperception of sleep. However, Harvey et al. (2005) reported similar findings in *euthymic* individuals with BD, who tended to overestimate their SL (by 40.6 min on average) and underestimate their TST (by 1.3 h on average). Ritter et al. (2016) also reported that euthymic individuals with BD underestimated their TST significantly more than HC. These two studies hence suggested that other factors, beyond the current mood state, might also drive misperception of sleep, which is also observed during euthymia.

Based on the inconsistencies in previous studies of misperception of sleep in BD, we thus aimed to characterize:

1. The correlations between subjective and objective measures of sleep (duration, latency and efficiency) in euthymic individuals with BD and in HC,
2. The differences in the magnitude of misperception of sleep in individuals with BD as compared to HC, and,
3. The putative demographic and/or clinical factors that contribute to misperception of sleep, including BD subtype (type 1 and type 2).

We hypothesized that the correlations between subjective and objective measures of sleep would be lower in BD than in HC, that the magnitude of misperception of sleep would be higher in BD than in HC, and that some demographic and clinical factors, including current mood symptoms, would increase the magnitude of misperception of sleep.

## Materials and methods

### Population

Individuals with BD were recruited during a euthymic phase from an outpatient specialized clinical unit, affiliated to the University of Paris in France. HC were recruited from the general population through adverts. The original description of the study is available in a previously published article (Geoffroy et al. 2014). This particular study was performed through post hoc analyses of available data and in a larger sample, and the participants were blind to the hypotheses of this study about sleep misperception.

Inclusion criteria for cases were: (1) age ≥ 18 years old; (2) DSM-IV diagnosis of BD as assessed using the Diagnostic Interview for Genetic Studies (DIGS) (Nurnberger et al. 1994); (3) euthymia for at least 3 months prior to study entry, defined as a score < 8 on both the Montgomery-Asberg Depression Rating Scale (MADRS) (Montgomery and Åsberg 1979) and the Young Mania Rating Scale (YMRS) (Young et al. 1978); (4) willingness and ability to give written informed consent for participation.

Exclusion criteria were: (1) being hospitalized and/or changes in the prescribed psychotropic medications during the 3 months prior to study; (2) receiving medical treatment for a condition known to alter sleep patterns; (3) current alcohol or illicit substance use disorder; (4) a diagnosis of a sleep disorder being confirmed through a sleep laboratory exam (obstructive sleep apnea, narcolepsy, restless leg syndrome and primary insomnia) lead to exclusion (however, the presence of symptoms of obstructive sleep apnea was not an exclusion criterion); (5) being involved in shift work, recent trans-meridian travel (> 3 h of jet-lag) or self-report of a major life event likely to disrupt sleep continuity (e.g. current pregnancy, recent child birth or recent bereavement).

Healthy controls (HC) were eligible for inclusion if they gave written informed consent and if a DIGS assessment confirmed an absence of a personal history of any DSM-IV disorders and an absence of a family history of schizophrenia, affective disorders and/or suicide attempts.

### Subjective measures of sleep

The subjective measures of sleep were obtained from the first four items of the PSQI, where participants are asked to estimate sleep duration, latency and total time in bed. The questionnaire was completed by all participants at the end of the actigraphy-recording period (see below), with instruction to estimate average values during the 21 past days of actigraphy-recording. TST was described in hours and SL in minutes. SE was calculated by dividing TST on total time in bed, then multiplied with 100 and described in percentage.

### Objective measures of sleep

Participants were asked to wear an actiwatch (Actiwatch AW-7, CamNtech^®^) on the wrist of their non-dominant hand for 21 consecutive days and to press the event-button on the actiwatch when they intended to sleep at night and when they woke up in the morning. Activity levels were scored in 60-second epochs. Data obtained from the recording were analysed by the Actiwatch software (Actiwatch Activity & Sleep Analysis Ltd CamNtechs 7.28, Cambridge, UK). Mean values over the 21 days period for TST, SL and SE were used for subsequent analyses.

### Calculation of misperception of sleep

The misperception of sleep for TST, SL and SE were operationalized as the absolute values of the differences between subjective and objective measures (i.e.,|PSQI value minus actigraphy value|). Misperception of TST were given in hours, SL in minutes, and SE in percentage.

### Demographic and clinical variables

Basic demographic data were collected at baseline. Diagnosis was made by experienced psychologists or psychiatrists, using structured interview with accordance to DSM-IV criteria (DIGS) (Nurnberger et al. 1994). Current depressive symptoms were assessed with the MADRS (Montgomery and Åsberg 1979), current manic symptoms were assessed with the YMRS (Young et al. 1978). The Berlin Questionnaire (Netzer et al. 1999) was used to investigate the presence of symptoms (snoring, quit breathing, tiredness and fatigue) and risk factors (high body mass index, high blood pressure) that are associated with the risk of presenting with obstructive sleep apnea (OSA), and thus assess a risk of OSA. Daily intake of coffee, alcohol and cigarettes were reported in a diary by the participants during the 21-day period of actigraphy, and the mean of these values were calculated and then used as a measure of daily use of these three substances.

### Statistics

Statistical analyses were carried out in SPSS V26 (IBM Corp). The assumption of normal distributions of misperception of sleep for TST, SL and SE were tested using Shapiro-Wilks test and the values were log-transformed for these three parameters to fit a normal distribution. Correlations between continuous variables were tested using Pearson correlation tests for normally distributed data, and otherwise using Spearman correlation tests. Differences between groups were tested using Chi square tests for categorical variables and t-tests for continuous variables, with further adjustment for potential confounders using analysis of covariance (ANCOVA). Multivariable regression analyses were used to test for the associations between measures of misperception of sleep (defined as dependent variables in three regression models) and demographic and clinical factors (defined as independent variables). Preliminary analyses and the distribution of the residuals of the regression models were examined with Predicted Probability (P–P) plots, histograms and scatterplots to ensure no violation of the assumptions of normality, linearity, multicollinearity and homoscedasticity. The stepwise procedure of the regression analysis was reserved for exploration if the entered models did not fit well.

Post-hoc analyses were also performed to test any differences between individuals with BD type 1 and with BD type 2.

## Results

### Sociodemographic data and sleep variables in cases and controls

A sample of 196 participants was included in the study, of which 133 individuals had BD (type 1 n = 100, type 2 n = 31, not otherwise specified = 2) and 63 were HC. Demographic data are given in Table 1. Individuals with BD were older and had a higher BMI as compared to HC. Significantly more cases were classified as at risk for OSA as compared to controls (p < .001). Although present at very low levels, depressive and manic symptoms were significantly higher in cases as compared to controls (both p < .001).

**Table 1:** Univariable comparisons between individuals with BD and HC for demographics, clinical and sleep variables

Variables	HC (n = 63)	BD (n = 133)	P-values
Age (mean)	36.24 (SD 13.54)	45.95 (SD 13.03)	.000
Sex (% female)	58.7% (n = 37)	61.7% (n = 82)	.695^a^
BMI	23.48 (SD 3.67)	25.64 (SD 4.51)	.001
OSA risk	3.2% (n = 2)	23.3% (n = 31)	.000^a^
Average daily coffee intake	1.31 (SD 1.10)	2.43 (SD 2.30)	.000
Average daily alcohol units	1.09 (SD 1.14)	1.00 (SD 1.26)	.634
Average daily cigarettes	2.06 (SD 4.12)	5.44 (SD 8.02)	.000
MADRS	.25 (SD 1.43)	2.29 (SD 3.44)	.000
YMRS	.07 (SD 0.33)	.77 (SD 1.55)	.000
TST by PSQI (hours)	7:07 (SD 1:00)	7:26 (SD 1:23)	.098
TST by actigraphy (hours)	7:46 (SD 0:43)	8:07 (SD 1:01)	.018
SL by PSQI (minutes)	18.83 (SD 16.86)	23.08 (SD 20.34)	.152
SL by actigraphy (minutes)	15.37 (SD 19.47)	15.10 (SD 13.43)	.910
SE by PSQI (in %)	90.57 (SD 7.88)	86.58 (SD 12.44)	.021
SE by actigraphy (in %)	84.31 (SD 8.07)	84.13 (SD 6.37)	.872
TST misperception^b^	0.86 (SD 0.36)	0.93 (SD 0.42)	.202
SL misperception^b^	3.36 (SD 2.05)	3.54 (SD 1.90)	.550
SE misperception^b^	2.72 (SD 1.19)	2.95 (SD 1.31)	.245

*HC* healthy controls, *BD* bipolar disorder, *BMI* body mass index, *OSA risk* any positive score related to obstructive sleep apnea on The Berlin Questionnaire, *MADRS* Montgomery and Asberg Depression Rating Scale, *YMRS* Young Mania Rating Scale, *PSQI* Pittsburgh Sleep Quality Index, *TST* total sleep time, *SL* sleep latency, *SE* sleep efficiency

^a^p values from Chi Square; ^b^log-transformed values given

Individuals with BD had a mean age of onset of 25.28 years (SD 8.86), the mean duration of illness was 20.42 years (SD 11.18), and the mean number of lifetime mood episodes was 7.29 (SD 4.80). About 41% of individuals with BD had a history of attempted suicide, with a mean number of suicidal attempts of 1.98 (SD 1.54). Individuals with BD type 1 had a higher coffee intake (in cups per day, 2.71 (SD 2.49) vs. 1.64 (SD 1.26), p = .023) than individuals with BD type 2, while none of the other variables listed in Table 1 significantly differed between BD subtypes (data not shown, available on request upon the authors).

Regarding TST, SL and SE (both subjective and objective measures), the unadjusted comparisons between cases and controls are presented in Table 1. After adjustment for age, BMI, OSA risk, coffee and tobacco intake, MADRS and YMRS scores, individuals with BD had a significantly longer TST as measured by both PSQI and actigraphy compared to HC (ANCOVA: p = .009 for PSQI measures and p = .004 for actigraphy). The differences between HC and BD were not significant after adjustment for the same covariates regarding SL (ANCOVA: p = .858 for PSQI measures and p = .797 for actigraphy), nor SE (ANCOVA: p = .478 for PSQI measures and p = .481 for actigraphy).

### Correlations between subjective and objective measures of sleep in cases and controls

Subjective and objective measures of TST were statistically significantly associated, with medium effect sizes in both groups (rho = .539, p < .001 in BD and rho = .584, p < .001 in HC). Subjective and objective measures of SL were statistically significantly associated, with a small effect size in BD (rho = .190, p = .029), but not in HC (rho = .125, p = .330). There was a trend-level association between subjective and objective measures of SE in both groups (rho = .166, p = .057 in BD and rho = .222, p = .081 in HC). The correlation coefficients were not significantly different in BD and in HC for any of the three sleep parameters (TST: p = 0.67; SL: p = 0.66; SE: p = 0.71).

When further investigating BD type 1 and BD type 2 separately, correlations between subjective and objective measures were as follows: TST: rho = .449, p < .001 in BD type 1 and rho = .750, p < .001 in BD type 2; SL: rho = .264, p = .104 in BD type 1 and rho = .175, p = .345 in BD type 2; SE: rho = .174, p = .083 in BD type 1 and rho = .329, p = .071 in BD type 2. The correlation coefficients were significantly different in BD type 1 and BD type 2 for TST (p = 0.02), but not for SL (p = 0.65) or SE (p = 0.44).

In the whole sample (cases with BD and HC), we also investigated whether the magnitude of sleep misperception was (un)related to the self-reported sleep parameters (TST/SL/SE). Data are presented in Additional file 1: Table S1. Results showed that the shorter the self-reported TST, the higher the misperception of TST (rho = −.466 p = .000). Furthermore, the higher the self-reported SL, the higher the misperception of SL (rho = .723 p = .000). There was no correlation between the self-reported SE and misperception of SE (p = .096) (see Additional file 1: Table S1 for details).

### Comparisons of misperception of sleep between cases and controls

Measures of misperception of sleep did not differ between HC and BD before and after adjustment for age, BMI, OSA risk, coffee and tobacco intake, MADRS and YMRS scores (see Table 1).

Regarding BD subtype, individuals with BD type 1 had a significantly higher level of misperception of SE as compared to individuals with BD type 2 (3.11 (SD 1.34) vs 2.38 (SD 1.11), p = .006), with no difference regarding misperception of TST and SL between BD type 1 and BD type 2 (see Additional file 1: Table S2).

### Factors associated with misperception of sleep in cases and controls

Since we found no difference in misperception of sleep between individuals with BD and HC, we conducted an analysis in the whole sample (both cases and controls) to test for associations with individual characteristics of interest (Table 2).

**Table 2:** Associations between misperception of sleep and demographic and clinical variables

Continuous variables	TST misperception		SL misperception		SE misperception	
	Rho	P	Rho	P	Rho	P
Age	.160	.025	.143	.046	.056	.433
MADRS	.142	.047	.189	.008	.160	.025
YMRS	.019	.792	.103	.152	.063	.379
Daily coffee intake	.025	.733	.000	.999	.045	.531
Daily alcohol intake	−.078	.275	−.161	.024	.089	.216
Daily cigarette intake	−.009	.902	−.016	.828	.021	.773

Categorical variables	*p* value (t-test)	p-value (t-test)	p-value (t-test)
Sex	.067	.214	.497
OSA risk	.001	.215	.420

*MADRS* Montgomery and Asberg Depression Rating Scale, *YMRS* Young’s Mania Rating Scale, *OSA risk* any positive score related to obstructive sleep apnea on The Berlin Questionnaire, *TST* total sleep time, *SL* sleep latency, *SE* sleep efficiency

We found statistically significant correlations between higher age and misperception of TST and SL; between a higher MADRS scores and misperception of TST, SL and SE; and between a lower alcohol intake and misperception of SL. OSA risk was associated with a higher misperception of TST. When investigating these correlations separately in HC and individuals with BD, we found that MADRS score and sex was significantly correlated with misperception of SL in HC, and that age, sex and OSA risk was significantly correlated with misperception of TST in individuals with BD (Additional file 1: Tables 3 and 4). The correlations analysed separately in individuals with BD type 1 and BD type 2 are further reported in detail in Additional file 1: Tables 5 and 6.

We then included age, sex, diagnosis (BD versus HC), OSA risk, MADRS score and current alcohol use (in units per day) as independent variables in three linear regression models, with misperceptions of TST, SL and SE as the dependent variables (Table 3).

**Table 3:** Results of the linear regression analyses for misperception of TST, SL and SE, and variables of interest

	Misperception of TST			Misperception of SL			Misperception of SE		
	B (SE)	β	P-value	B (SE)	β	P-value	B (SE)	β	P-value
Age	.004 (.002)	.144	.052	.021 (.010)	.152	.043	–	–	–
Sex	–	–	–	–	–	–	–	–	–
OSA risk	.217 (.077)	.203	.005	–	–	–	–	–	–
MADRS	.017 (.009)	.133	.070	.100 (.046)	.159	.032	–	–	–
Alcohol intake		–	–	−291 (.117)	−.183	.014	–	–	–
Diagnosis	–	–	–	–	–	**–**	**–**	**–**	**–**
Total model performance	R^2^ = .108, F Change = 3.799, Sig.F Change = .001			R^2^ = .086, F Change = 2.949, Sig.F Change = .009			R^2^ = .024, F Change = .749, Sig.F Change = .611		

All independent variables entered, only significant and trend-significant values given in table

*MADRS* Montgomery and Asberg Depression Rating Scale, *OSA risk* any positive score related to obstructive sleep apnea on The Berlin Questionnaire

Diagnosis was not associated with any of the misperception measures. Age was associated with misperception of TST (at a trend level) and SL. OSA risk and MADRS score (at a trend level) were associated with misperception of TST. MADRS score and alcohol units per day were associated with misperception of SL.

The model with misperception of SE as the dependent variable did not fit well when all factors were entered, and did not retain any associated factors after the stepwise procedure. However, because we reported a difference in misperception of SE when comparing individuals with BD type 1 and type 2, we further divided the diagnosis variable into HC, BD type 1 and BD type 2 by the use of dummy-variables, and entered these variables in the model. The stepwise model then only retained the dummy-variable with BD type 1 as significantly associated with misperception of SE (B = .492 (SE = .179), β = .193, p = .007) and the whole model fitted moderately well (R^2^ = .037, F Change = 7.531, Sig. F Change = .007). Since no differences in misperception of TST and SL were observed between BD type 1 and BD type 2, we provided no further analyses stratified on BD subtype.

## Discussion

The correlations between subjective and objective measures of sleep parameters did not differ significantly between individuals with BD and HC. As a whole, the correlations between subjective and objective measures seem to be more evident with regards to TST (suggesting lower misperception), and less so to SL and SE (suggesting more misperception) in both groups. As such we did not replicate findings from previous studies (Gonzalez et al. 2013; Boudebesse et al. 2014; Krishnamurthy et al. 2018). In line with this, we did not find any differences between individuals with BD and HC for any of the three misperception of sleep estimates. These findings thus did not confirm results of some previous studies that have compared individuals with BD and HC (Krishnamurthy et al. 2018; Harvey et al. 2005; Ritter et al. 2016).

These discrepancies between our study and the available literature deserve some comments. First, the number of participants included in previous studies were relatively low (lower than 54 individuals in total), and may be chance findings due to random sampling fluctuations. Second, two studies included so called ‘symptomatic individuals with BD’ (i.e. with at least moderate to severe depressive symptoms or clinically significant manic symptoms) (Gonzalez et al. 2013; Krishnamurthy et al. 2018). Thus, as noted by the authors, current mood symptoms might have been the basis for the observed differences, which is in line with our findings of an effect of depressive symptoms on misperception. Two studies, however, included individuals with BD during a *euthymic* phase (Harvey et al. 2005; Ritter et al. 2016) and, nonetheless, suggested a higher misperception of sleep in BD. Harvey et al. (2005) found a tendency for misperception of sleep, while Ritter et al. (2016) found underestimation of TST, but no overestimation of SL, in individuals with BD. However, no adjustment for residual depressive symptoms was proposed in either study. Third, when considering the study by Harvey et al. (2005) showing misperception of sleep in euthymic individuals with BD type 1, more than half of the patients had a diagnosis of insomnia and about two-thirds had a sleep disorder, whereas the control group were ‘good sleepers’. The over-representation of insomnia and sleep disorders in this particular sample may have driven the differences observed as compared to ‘good sleepers’. Fourth, previous studies had a short duration of actigraphy recording (from 5 to 8 days as compared to 21 days in our study), which may be too short to provide a valid and stable estimation of objective sleep parameters. Indeed, short measurement periods with actigraphy may be more sensitive to random variations, thus the longer the measurement period, the more reliable the measure (Tryon 2004). Finally, when comparing our results to the existing literature, it is worth mentioning that the studies by Ritter et al. (2016) and Gonzalez et al. (2013) both used prospective sleep diaries as measures of subjective sleep. Boudebesse et al. (2014) and Krishnamurthy et al. (2018) used retrospective questionnaires (PSQI), while Harvey et al. (2005) used both prospective sleep diaries and retrospective questionnaires (PSQI). Given potential recall bias, a clinical questionnaire that assesses the “last month” such as the PSQI might potentially be more prone for misperception than a questionnaire that assesses the “last 7 days”. Direct comparisons between these studies may therefore be limited by the different methodology in approaching subjective measures of sleep, and is to be further discussed in the limitations section below.

In the last part of our analyses, we obtained findings suggesting that a few demographic and clinical factors were associated with misperception of sleep, independently of the clinical diagnosis. Older age was associated with misperception of TST and SL. The contribution of age may be due to age-related differences in perception of time in general (Wittmann and Lehnhoff 2005). In this context, age at onset and duration of illness did not correlate to misperception of sleep in the BD sample (data not shown, available on request), hence suggesting that the impact of age is not a proxy for the duration or chronicity of the disorder. Depressive symptoms, even at very low levels as seen in the current sample, also contributed to misperception of SL and to a lesser extent to misperception of TST. Misperception of SL has been observed in major depressive disorders in previous studies (Rotenberg et al. 2000; Armitage et al. 1997), and this study hence adds further arguments that current mood states may be a driver of misperception of sleep in individuals not only with BD, but also in HC, and potentially in other groups with prevalent depressive symptomatology. OSA risk was also associated with misperception of TST. OSA risk is very frequent in BD, and is linked to sleep fragmentation (Basit 2020; Geoffroy et al. 2019), that in turn may affect an individuals’ perception of sleep, and thus explain the observed association. Of interest, and quite surprisingly, increased average alcohol intake per day contributed to a lower magnitude of misperception of SL. In a previous study, we did not find any associations between alcohol use and SL (Gross et al. 2020). This intriguing result would deserve replication.

Finally, when we included BD subtype in the analyses, we found that misperception of SE may differ between BD type 1 compared to BD type 2. This finding was the only specific finding with regards to BD. This should, however, be interpreted with caution since exploratory and post hoc analyses and we cannot exclude that this represents a chance finding.

The main strengths of the current study were (1) its large number of participants for an actigraphy study, (2) the euthymic state of the individuals with BD, and (3) the length of actigraphy recording. Nevertheless, there are also several methodological limitations to discuss. First, we used retrospective data from the PSQI, to assess subjective measures of sleep after the period of measurement with actigraphy. This measure can be hampered by recall bias and averaging, and could thus increase inaccuracies as compared to daily recording (e.g. sleep diaries), which may be more accurate. However, as Krishnamurthy et al. also reported, the collection of sleep information retrospectively is reflecting common clinical practice, and should therefore be considered closer to the “real world” setting (Krishnamurthy et al. 2018). On the other hand, wearing the actiwatch for 21 days may have worked as a reminder to pay more close attention to the qualitative and quantitative aspects of sleep, and thus may result in a more accurate subjective reporting than could otherwise be expected in a clinical setting. Second, we used the absolute values of the differences between subjective and objective measures to assess the magnitude of misperception. This may hide some nuances, such as discriminating between over- and underestimation. Third, all individuals with BD currently used psychotropic medications, challenging the interpretation of what role the medication may have on sleep perception (as compared to drug naïve or untreated cases). Fourth, we did not screen participants for symptoms of insomnia, as they did in the study by Harvey et al. (2005). This means that we were unable to assess whether symptoms of insomnia also contribute to sleep misperception. This has been highlighted by Harvey, who observed sleep misperception within a sample of individuals with BD, out of which more than half reported insomnia symptoms. Finally, this study was exploratory, and corrections for multiple testing was not applied.

For clinicians, the assessment of sleep complaints and disturbances is crucial in BD because of their high prevalence and possible links to the outcome of the disorder in terms of affective and cognitive functioning, quality of life, metabolic health and relapse rates (Harvey et al. 2009; Gruber et al. 2009; Eidelman et al. 2010; Gruber et al. 2011; Etain et al. 2017; Brochard et al. 2018). Given the previous articles suggesting that patients with BD might present with greater sleep misperception as compared to non-clinical populations, clinicians may doubt the validity of questionnaires and/or self-reported diaries. As actigraphy is not easily accessible in daily clinical practice, this may not be a viable option in every case. This study suggests that self-assessment of the main sleep parameters (TST, SL and SE) with the PSQI is feasible in individuals with BD, and not significantly biased because of sleep misperception. However, since sleep misperception was associated with mild depressive symptoms, older age, or OSA risk, the reliability of such subjective measures (here the PSQI) may decrease as the misperception of sleep increases. The correlate is that, among older individuals, with more depressive symptoms and higher risk of OSA, actigraphy should be preferred to provide a more reliable assessment of the sleep parameters. For individuals who are younger, with a low level of depressive symptoms and a low risk of OSA, the use of the PSQI may be clinically relevant and sufficiently reliable to estimate sleep duration, latency and efficiency. Hence, this study may guide the choice of the optimal method for the assessment of sleep in clinical practice and further recommend the use of actigraphy in sub-populations that are more prone to sleep misperception. We found no evidence that a diagnosis of BD per se should be regarded as such. If sleep misperception is identified in a given individual, it might then be the target for future psychosocial and educational interventions about sleep. For instance, the perception of low sleep quality may be exaggerated by an overestimation of SL and an underestimation of TST, which may lead to additional stress and a feeling of inability to sleep. In this case, cognitive behavior therapy may be a useful tool to improve subjective–objective sleep discrepancy, as previously suggested in patients with chronic forms of insomnia (Crönlein et al. 2019).

## Conclusion

In conclusion, our study suggests that several factors such as age, OSA risk, in addition to current residual depressive symptoms, are the main contributors to misperception of sleep in BD. A diagnosis of BD (in euthymic phase) is not per se associated with misperception of sleep. Our findings implicate that a subjective measure of sleep with the PSQI is feasible for clinical use in euthymic individuals with BD. The use of objective measuring methods should therefore be prioritized, if available, in patients who present with these risk factors of misperception of sleep.

In future studies it would be of interest to investigate more closely the relevance of discriminating between under- and overestimation of sleep parameters, as the difference between the two may represent separate misperception entities, and may associate differently to diagnosis and other variables of interest. Another area of interest is to determine whether the factors that correlate to sleep misperception differ or change based on BD subtype, presence of insomnia or other stratification models.

## Supplementary information

**Additional file 1: Table S1.** Correlations between self-rated TST, SL and SE (measured by the PSQI) and sleep misperception variables. **Table S2.** Comparisons between individuals with BD type 1 and BD type 2 for demographics, clinical and sleep variables. Individuals with BD type not otherwise specified were excluded (n=2). **Table S3.** Associations between misperception of sleep and demographic and clinical variables in HC (n=63). **Table S4.** Associations between misperception of sleep and demographic and clinical variables in individuals with BD (n=133). **Table S5.** Associations between misperception of sleep and demographic and clinical variables in individuals with BD type 1 (n=100). **Table 6.** Associations between misperception of sleep and demographic and clinical variables in individuals with BD type 2 (n=31).

## Data Availability

The datasets used and/or analysed during the current study are available from the corresponding author on reasonable request.

## Abbreviations

BD: Bipolar disorder
BMI: Body mass index
DIGS: Diagnostic interview for genetic studies
HC: Healthy controls
MADRS: Montgomery and asberg depression rating scale
OSA: Obstructive sleep apnea
PSQI: Pittsburgh sleep quality index
SE: Sleep efficiency
SL: Sleep latency
TST: Total sleep time
YMRS: Young’s mania rating scale
