# Supplementary material for: Misperception of sleep in bipolar disorder: an exploratory study using questionnaire versus actigraphy
Source: Int J Bipolar Disord. 2020 Nov 20;8:34. doi: 10.1186/s40345-020-00198-x (PMC7677419; doi:10.1186/s40345-020-00198-x)
Supplement: Supplementary file 1 — Additional file 1: Table S1. Correlations between self-rated TST, SL and SE (measured by the PSQI) and sleep misperception variables. Table S2. Comparisons between individuals with BD type 1 and BD type 2 for demographics, clinical and sleep variables. Individuals with BD type not otherwise specified were excluded (n=2). Table S3. Associations between misperception of sleep and demographic and clinical variables in HC (n=63). Table S4. Associations between misperception of sleep and demographic and clinical variables in individuals with BD (n=133). Table S5. Associations between misperception of sleep and demographic and clinical variables in individuals with BD type 1 (n=100). Table 6. Associations between misperception of sleep and demographic and clinical variables in individuals with BD type 2 (n=31). [file 40345_2020_198_MOESM1_ESM.pdf]

<u>Supplementary table 1:</u> *Correlations between self-rated TST, SL and SE (measured by the PSQI) and sleep misperception variables.*

	**TST misperception**		**SL misperception**		**SE misperception**	
**PSQI items**	rho	p	rho	p	rho	p
TST	-.466	.000	-.144	.113	-.057	.430
SL	.275	.000	.723	.000	.125	.081
SE	-.533	.000	-.284	.000	-.119	.096

TST=total sleep time; SL=sleep latency; SE=sleep efficiency.PSQI: Pittsburg Sleep Quality Index

<u>Supplementary table 2:</u> *Comparisons between individuals with BD type 1 and BD type 2 for demographics, clinical and sleep variables. Individuals with BD type not otherwise specified were excluded (n=2).*

**Variables**	**BD 1 (n=100)**	**BD 2 (n=31)**	**p-values**
Age (mean)	45.16 (SD 12.82)	48.62 (SD 13.97)	.201
Sex (% female)	59.0% (n=59)	67.7% (n=21)	.363*
BMI	25.63 (SD 4.46)	25.49 (SD 4.83)	.886
OSA risk	20.0% (n=20)	32.3% (n=10)	.247*
Average daily coffee intake	2.71 (SD 2.49)	1.64 (SD 1.26)	.023
Average daily alcohol units	1.08 (SD 1.38)	.82 (SD .82)	.323
Average daily cigarettes	5.85 (SD 8.36)	4.47 (SD 6.98)	.406
MADRS	2.31 (SD 3.25)	2.32 (SD 4.12)	.986
YMRS	.72 (SD 1.54)	.84 (SD 1.59)	.711
TST by PSQI (hours)	7:31 (SD 1:23)	7:16 (SD 1:23)	.376
TST by actigraphy (hours)	8:08 (SD 1:02)	8:03 (SD 1:02)	.684
SL by PSQI (minutes)	22.10 (SD 20.99)	25.31 (SD 18.44)	.446
SL by actigraphy (minutes)	14.43 (SD 12.30)	16.97 (SD 16.79)	.362
SE by PSQI (in %)	86.77 (SD 13.48)	85.76 (SD 8.57)	.693
SE by actigraphy (in %)	84.19 (SD 6.61)	83.93 (SD 5.84)	.843
TST misperception**	0.93 (SD 0.45)	0.93 (SD 0.32)	.987
SL misperception**	3.58 (SD 1.92)	3.35 (SD 1.91)	.563
SE misperception**	3.11 (SD 1.34)	2.38 (SD 1.11)	.006

*: p values from Chi Square; **: log-transformed values given; HC=healthy controls; BD=bipolar disorder; BMI=body mass index; OSA risk=any positive score related to obstructive sleep apnea on The Berlin Questionnaire; MADRS=Montgomery and Asberg Depression Rating Scale; YMRS=Young Mania Rating Scale; PSQI=Pittsburgh Sleep Quality Index; TST=total sleep time; SL=sleep latency; SE=sleep efficiency.

Supplementary table 3: *Associations between misperception of sleep and demographic and clinical variables in HC (n=63).*

	**TST misperception**		**SL misperception**		**SE misperception**	
**Continuous variables**	rho	p	rho	p	rho	p
Age	.044	.734	.227	.074	-.051	.693
MADRS	.190	.136	**.295**	**.019**	.155	.227
YMRS	.175	.171	.168	.189	-.084	.514
Daily coffee intake	.062	.629	.003	.982	-.184	.150
Daily alcohol intake	-.130	.309	-.221	.082	.247	.051
Daily cigarette intake	.114	.373	-.083	.518	.068	.596
**Categorical variables**	p-value (t-test)		p-value (t-test)		p-value (t-test)	
Sex	.974		**.034**		.340	
OSA risk *	-		-		-	

MADRS=Montgomery and Asberg Depression Rating Scale; YMRS=Young's Mania Rating Scale; OSA risk=any positive score related to obstructive sleep apnea on The Berlin Questionnaire; TST=total sleep time; SL=sleep latency; SE=sleep efficiency.

* Analysis not performed since only 2 healthy controls had a 'high risk' of OSA

Supplementary table 4: *Associations between misperception of sleep and demographic and clinical variables in individuals with BD (n=133).*

	**TST misperception**		**SL misperception**		**SE misperception**	
**Continuous variables**	rho	p	rho	p	rho	p
Age	**.201**	**.020**	.016	.852	.066	.448
MADRS	.116	.184	.153	.078	.141	.105
YMRS	-.025	.772	.090	.304	.061	.487
Daily coffee intake	-.028	.748	-.020	.822	.093	.289
Daily alcohol intake	-.047	.591	-.120	.169	.047	.593
Daily cigarette intake	-.069	.431	-.005	.956	-.021	.811
**Categorical variables**	p-value (t-test)		p-value (t-test)		p-value (t-test)	
Sex	**.034**		.960		.871	
OSA risk	**.009**		.679		.655	

MADRS=Montgomery and Asberg Depression Rating Scale; YMRS=Young's Mania Rating Scale; OSA risk=any positive score related to obstructive sleep apnea on The Berlin Questionnaire; TST=total sleep time; SL=sleep latency; SE=sleep efficiency.

<u>Supplementary table 5:</u> *Associations between misperception of sleep and demographic and clinical variables in individuals with BD type 1 (n=100).*

	**TST misperception**		**SL misperception**		**SE misperception**	
**Continuous variables**	rho	p	rho	p	rho	p
Age	**.227**	**.023**	.057	.574	.020	.843
MADRS	.076	.455	.067	.508	.074	.466
YMRS	.033	.742	.188	.062	.121	.232
Daily coffee intake	-.021	.839	.062	.537	.074	.463
Daily alcohol intake	-.067	.510	-.099	.326	.018	.855
Daily cigarette intake	-.098	.333	.016	.871	.003	.973
**Categorical variables**	p-value (t-test)		p-value (t-test)		p-value (t-test)	
Sex	.073		.579		.659	
OSA risk	**.003**		.866		.368	

MADRS=Montgomery and Asberg Depression Rating Scale; YMRS=Young's Mania Rating Scale; OSA risk=any positive score related to obstructive sleep apnea on The Berlin Questionnaire; TST=total sleep time; SL=sleep latency; SE=sleep efficiency.

<u>Supplementary table 6:</u> *Associations between misperception of sleep and demographic and clinical variables in individuals with BD type 2 (n=31).*

	**TST misperception**		**SL misperception**		**SE misperception**	
**Continuous variables**	rho	p	rho	p	rho	p
Age	.070	.710	-.092	.621	.301	.100
MADRS	.311	.089	**.422**	**.018**	**.355**	**.050**
YMRS	-.211	.254	-.139	.454	-.092	.623
Daily coffee intake	.030	.873	-.223	.227	-.037	.843
Daily alcohol intake	.107	.566	-.130	.485	.148	.427
Daily cigarette intake	.095	.612	-.034	.854	-.223	.227
**Categorical variables**	p-value (t-test)		p-value (t-test)		p-value (t-test)	
Sex	.344		.351		.864	
OSA risk	.921		.299		.765	

MADRS=Montgomery and Asberg Depression Rating Scale; YMRS=Young's Mania Rating Scale; OSA risk=any positive score related to obstructive sleep apnea on The Berlin Questionnaire; TST=total sleep time; SL=sleep latency; SE=sleep efficiency.
